# A structurally-characterized peroxomanganese(iv) porphyrin from reversible O_2_ binding within a metal–organic framework†
†Electronic supplementary information (ESI) available: Additional experimental, spectroscopic, gas adsorption, and crystallographic data. Crystallographic information files for **1** and **2** can be obtained from the Cambridge Structural Database. CCDC 1570584 and 1570585. For ESI and crystallographic data in CIF or other electronic format see DOI: 10.1039/c7sc03739b


**DOI:** 10.1039/c7sc03739b

**Published:** 2017-12-14

**Authors:** Audrey T. Gallagher, Jung Yoon Lee, Venkatesan Kathiresan, John S. Anderson, Brian M. Hoffman, T. David Harris

**Affiliations:** a Department of Chemistry , Northwestern University , 2145 Sheridan Road , Evanston , IL 60208-3113 , USA . Email: dharris@northwestern.edu

## Abstract

The role of peroxometal species as reactive intermediates in myriad biological processes has motivated the synthesis and study of analogous molecular model complexes. Peroxomanganese(iv) porphyrin complexes are of particular interest, owing to their potential ability to form from reversible O_2_ binding, yet have been exceedingly difficult to isolate and characterize in molecular form. Alternatively, immobilization of metalloporphyrin sites within a metal–organic framework (MOF) can enable the study of interactions between low-coordinate metal centers and gaseous substrates, without interference from bimolecular reactions and axial ligation by solvent molecules. Here, we employ this approach to isolate the first rigorously four-coordinate manganese(ii) porphyrin complex and examine its reactivity with O_2_ using infrared spectroscopy, single-crystal X-ray diffraction, EPR spectroscopy, and O_2_ adsorption analysis. X-ray diffraction experiments reveal for the first time a peroxomanganese(iv) porphyrin species, which exhibits a side-on, η^2^ binding mode. Infrared and EPR spectroscopic data confirm the formulation of a peroxomanganese(iv) electronic structure, and show that O_2_ binding is reversible at ambient temperature, in contrast to what has been observed in molecular form. Finally, O_2_ gas adsorption measurements are employed to quantify the enthalpy of O_2_ binding as *h*_ads_ = –49.6(8) kJ mol^–1^. This enthalpy is considerably higher than in the corresponding Fe- and Co-based MOFs, and is found to increase with increasing reductive capacity of the M^II/III^ redox couple.

## Abstract 2

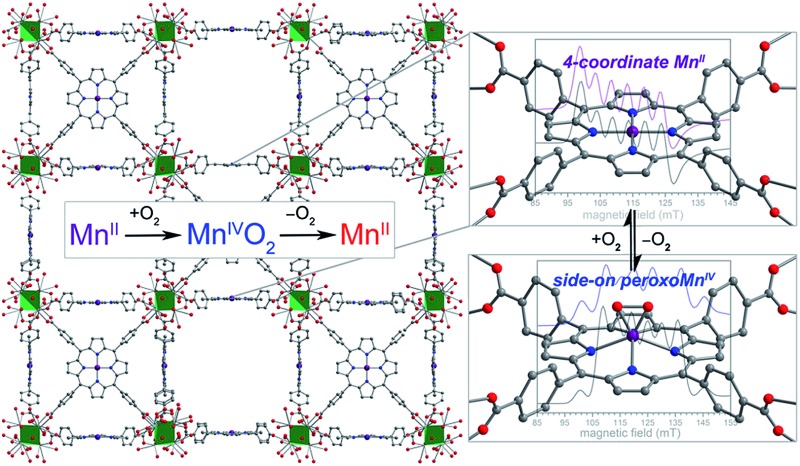
Within a MOF, a side-on peroxomanganese(iv) porphyrin has been isolated and comprehensively examined.

## Introduction

The activation of O_2_ by metalloproteins is central to a wide range of biological processes, including bond activation, O_2_ transport, metabolism, and the regulation of reactive oxygen species.^1^ In many of these processes, metal complexes of O_2_^2–^, or peroxide, represent reactive intermediates that are generated during the catalytic cycles of enzymatic reactions.1a–e,^2^ For instance, C–H bond activation by heme-containing enzymes involves peroxoiron intermediates.1a–c,2a,b,d In addition, water oxidation by the oxygen-evolving complex in photosystem II2*e* and the degradation of superoxide ion by manganese superoxide dismutase2*a* are postulated to proceed through peroxomanganese intermediates. The prominence and unusual reactivity of peroxoiron and manganese species in biology has motivated efforts to synthesize and characterize synthetic molecular model complexes, largely in order to elucidate the structure, physical properties, and chemical reactivity of these intermediates.2b,^3^ Indeed, tremendous progress has been made in the synthesis and study of both peroxoiron and manganese complexes. For instance, a number of mononuclear heme^4^ and non-heme^5^ peroxoiron complexes have been isolated, including two examples5a,b that have been structurally characterized. In addition, several mononuclear peroxomanganese species have been isolated,^6^–^8^ with two recent structurally-characterized examples featuring Mn^IV^.^9^

Despite these advances, significant challenges remain in the isolation of peroxomanganese complexes that display reversible O_2_ binding under ambient conditions, an important function often found in biological systems.^10^ Toward this end, manganese(ii) porphyrin complexes present an attractive platform, owing to their ability to carry out the two-electron reductive activation of O_2_ (ref. 11) and thus form high-valent peroxomanganese complexes.^12^ To date, one crystal structure of an O_2_ coordinated to a manganese porphyrin has been reported. This complex, formed by addition of KO_2_ to (TPP)Mn^II^ (H_2_TPP = 5,10,15,20-tetraphenylporphyrin), was shown to feature a Mn^III^ ion coordinated to a peroxo ligand in a side-on, η^2^ binding mode.7*a* While [(TPP)Mn(O_2_)]^–^ provides an important structural example of the peroxo binding mode, the presence of Mn^III^ renders O_2_ loss unfavourable, as it would necessitate the formation of a high-energy Mn^I^ species. In contrast, peroxomanganese(iv) complexes, as formed by reaction of Mn^II^ with O_2_, have been probed by numerous spectroscopic techniques, including EPR,12a,c,e vibrational,12l,n NMR,12*k* and UV/visible12a,c spectroscopies, in addition to computational methods.12d,i Although these investigations support a side-on peroxomanganese(iv), this geometry has not been confirmed by a crystal structure. Furthermore, the thermal instability of peroxomanganese(iv) species has limited their characterization and hindered a thorough investigation of their structure and properties.

A key limitation in the study of O_2_ binding in molecular metalloporphyrins is the propensity for these species to form oxo-bridged complexes *via* irreversible bimolecular condensation reactions.^13^ For instance, in Fe^II^ and Co^II^ complexes, highly elaborate ligands such as the sterically protected “picket-fence” porphyrins were necessary to prevent bimolecular decomposition reactions and enable the isolation and thorough characterization of six-coordinate Fe and Co–O_2_ adducts, which feature axial imidazole or thiolate ligands.^14^ In contrast, analogous axially-ligated Mn^II^ complexes do not bind O_2_, which has been attributed to the preference for five-coordinate geometries in porphyrinic Mn ions.12b,g Consequently, no peroxomanganese(iv) porphyrin species have been isolated or studied under ambient conditions.

As an alternative to employing molecular systems, one can envision isolating a peroxomanganese(iv) complex within a porphyrinic metal–organic framework (MOF). Here, the porous, solid-state structure of the MOF prevents bimolecular condensation reactions and enables introduction of gas-phase substrates in the absence of exogenous solvent. Furthermore, whereas the inherent reactivity of the peroxomanganese(iv) complexes has precluded crystallographic characterization, the crystallinity of MOFs provides an ideal platform to carry out single-crystal X-ray diffraction analysis. Illustrative of this approach, we have previously shown that the porphyrinic MOF PCN-224 (ref. 15) can be employed to study low-coordinate O_2_ adducts^16^,^17^ and labile carbonyl complexes.^18^ Herein, we comprehensively examine O_2_ binding to Mn^II^ in PCN-224 using a host of physical methods. Taken together, these experiments unambiguously establish the presence of a side-on peroxomanganese(iv) species, and the O_2_ binding is shown to be reversible even at ambient temperature.

## Results and discussion

### Synthesis of PCN-224Mn^II^

**Fig. 1:**
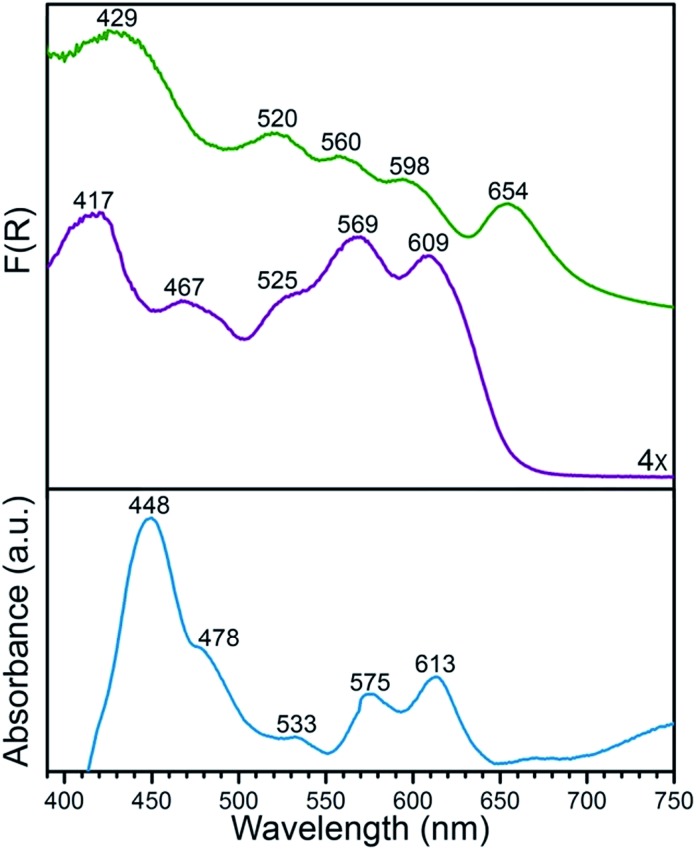
Solid-state diffuse reflectance UV/visible spectra for PCN-224 (green) and its Mn^ii^-metalated form **1** (purple), plotted as the Kubelka–Munk function F(R). For comparison, the absorption spectrum for a toluene suspension of **1** (blue) is shown, highlighting changes in the peak positions with and without toluene.

The porphyrinic MOF PCN-224 was synthesized as previously described.^15^ Subsequent metalation of the porphyrin with Mn^II^ was carried out by heating single crystals of PCN-224 under N_2_ in a DMF solution containing excess MnBr_2_ and 2,6-lutidine, followed by evacuation at 150 °C for 12 h, to give the compound PCN-224Mn^II^ (**1**). Complete metalation of the porphyrin within the bulk crystalline material was confirmed by solid-state diffuse reflectance UV/visible spectroscopy, trace metals analysis and powder X-ray diffraction (see Fig. 1 and S1 and Experimental section). Furthermore, N_2_ adsorption data collected for a desolvated sample of **1** at 77 K provided a Brunauer–Emmett–Teller surface area of 2455 m^2^ g^–1^ (see Fig. S2†), close to the accessible surface reported for other metalated variants of PCN-224,^15^–^17^,^19^ thereby confirming the retention of porosity upon metalation and the successful removal of solvent molecules from the pores. While several manganese porphyrin-containing MOFs have been reported, to our knowledge,^20^**1** represents the first example of a MOF that features a four-coordinate Mn^II^ porphyrin complex.

The diffuse reflectance UV/visible spectrum obtained for an activated sample of **1** exhibits similar peak maxima to the absorption spectrum of **1** suspended in toluene, but nevertheless features key differences (see Fig. 1). Most notably, the spectrum reported for **1** in toluene displays a Soret band at 448 nm, while the spectrum for activated **1** features a Soret band at 417 nm. These differences can possibly be attributed to the rigorous four-coordinate nature of the Mn center in **1**, compared to a slight distortion from local *D*_4h_ symmetry at Mn in the toluene solution imposed by Mn–toluene interactions as has been observed for the molecular analogue, (TPP)Mn (see crystallography discussion below).8b,^16^ More specifically, these changes may be attributed to the difference in mixing of metal- and porphyrin-based π orbitals in the two complexes, as changes in the degree of mixing are known to influence the resulting adsorption spectra of metalloporphyrin complexes.^21^ In the case of the rigorously in-plane Mn center, the maximal overlap of Mn d_*xz*,*yz*_ and porphyrin π* orbitals may account for the changes in the features in the electronic spectrum of **1**.

### Infrared spectroscopy

**Fig. 2:**
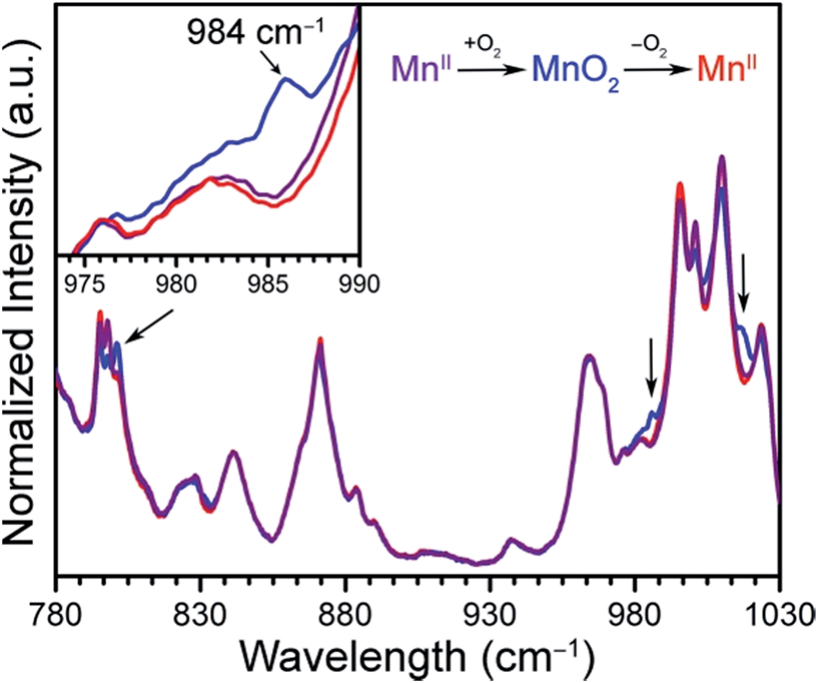
DRIFTS spectra for **1** at 298 K under static vacuum (purple), upon addition of O_2_ (blue), and after subsequent purging with Ar (red). The inset shows an expanded view of the spectra, highlighting the *ν*_O–O_ vibration of the dioxygen adduct in **2** at 984 cm^–1^.

As an initial investigation into the interaction between Mn^II^ and O_2_, a desolvated sample of **1** was exposed to *ca.* 1 atm of dry O_2_ and monitored by diffuse reflectance infrared Fourier transform spectroscopy (DRIFTS). The addition of O_2_ to **1** at 298 K was accompanied by the appearance of three new vibrations, situated at 801, 984, and 1017 cm^–1^ (see Fig. 2), indicative of conversion to a Mn–O_2_ complex in the compound PCN-224MnO_2_ (**2**). The vibrations at 801 and 1017 cm^–1^ have been previously attributed to a lowering of local symmetry from *D*_4h_ to *C*_2v_ at Mn^II^ upon O_2_ binding.12*l* This symmetry reduction occurs due to a displacement of Mn from the N_4_ plane of the porphyrin upon the addition of an axial ligand, in this case O_2_. Moreover, the feature at 984 cm^–1^ is identical within error to the formerly assigned *ν*_O–O_ vibration from a mixture of the molecular complex (TPP)Mn^II^ and O_2_, as isolated in a frozen Ar matrix at 15 K.12*l* Remarkably, in contrast to molecular systems, where the deoxymanganese(ii) complex was only partially regenerated after purging with N_2_ at –78 °C, similar purging here with dry Ar gas at 25 °C gave a spectrum identical to that obtained for **1** before adding O_2_ (see Fig. 2).12*c* This process of adding then removing O_2_ could be cycled at least three times, underscoring the ability of the solid-state MOF to enable reversible O_2_ binding (see Fig. S3†).

The *ν*_O–O_ vibration in **2** at 984 cm^–1^ is considerably lower than those of *ν*_O–O_ = 1195 and 1278 cm^–1^ previously observed for O_2_ adducts of (TPP)Fe and (TPP)Co at 15 K, which have been unambiguously assigned as superoxometal(III) species.^22^ This contrast suggests that O_2_ binding in PCN-224Mn^II^ involves a two-electron transfer from Mn^II^ to O_2_ to give a peroxomanganese(iv) species, rather than one-electron transfer to give a superoxomanganese(iii) species.12a–c,e–n Here, the weaker O–O bond in **2** relative to superoxo complexes can be primarily attributed to a doubly occupied π* orbital of an O_2_^2–^ ligand compared to singly occupied orbital of O_2_˙^–^. Additionally, the *ν*_O–O_ = 984 cm^–1^ in **2** is higher than those of *ν*_O–O_ = 806 cm^–1^ and 898 cm^–1^ previously reported for the peroxoiron(iii) complex3*a* [(OEP)FeO_2_]^–^and the peroxotitanium(iv) complex^23^ (OEP)TiO_2_ (H_2_OEP = 2,3,7,8,12,13,17,18-octaethylporphyrin), which both feature side-on coordination of O_2_^2–^. This difference may stem from increasing stabilization of the σ bond of O_2_^2–^ with increasing effective nuclear charge moving from Fe^III^ to Ti^IV^ to Mn^IV^. In addition, the concomitant increase in Lewis acidity across the series may serve to pull electron density out of the O_2_^2–^, thereby strengthening the O–O bond by alleviating electron–electron repulsion in O_2_^2–^. While the foregoing comparison of data is consistent with the presence of a side-on O_2_^2–^ in **2**, analysis of infrared spectra alone cannot definitively confirm this assignment.

### Single-crystal X-ray diffraction

**Fig. 3:**
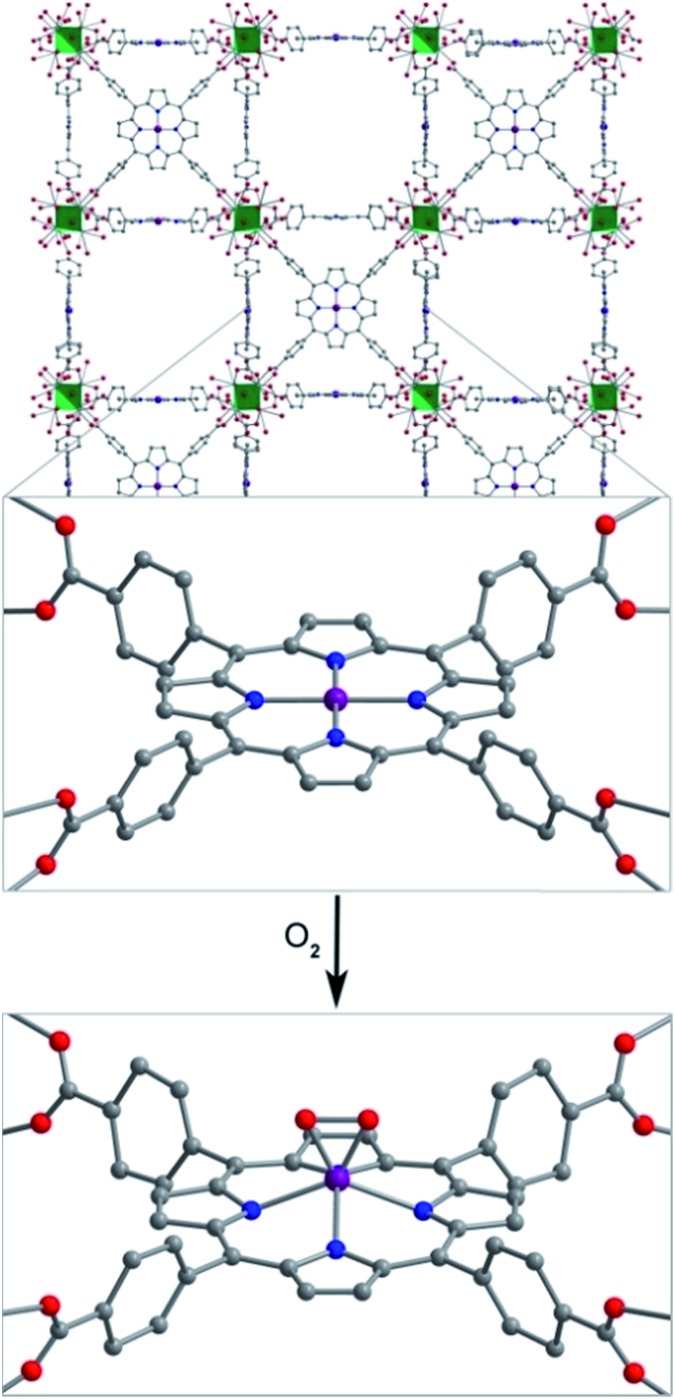
Reaction of **1** with O_2_ to form **2**. Vertices of the green octahedra represent Zr atoms; purple, red, blue, and gray spheres represent Mn, O, N, and C atoms, respectively; H atoms are omitted for clarity. Selected interatomic distances (Å) for **1**: Mn···N_4_ 0, Mn–N 1.998(5); for **2**: Mn···N_4_ 0.80(2), Mn–O 1.76(3), Mn–N 2.170(9).

The stability of the Mn–O_2_ adduct in **2** prompted us to investigate both **1** and **2** using single-crystal X-ray diffraction analysis. The structure of **1** exhibits a four-coordinate Mn^II^ center, residing in a square planar coordination environment on a crystallographic special position of *mm*2 site symmetry (see Fig. 3, S3, and Table S1†). Importantly, no significant residual electron density was located in the difference Fourier map, confirming the absence of axial ligation at Mn. The Mn–N distance of 1.998(5) Å is notably shorter than those of 2.082(2)–2.085(2) Å previously reported for the toluene-solvated compound (TPP)Mn·2C_7_H_8_.12b,^24^ While this molecular compound features a pseudo four-coordinate Mn^II^ center, weak contacts between Mn and a toluene molecule, with a closest Mn–C_toluene_ distance of 3.04 Å, lead to a 0.19 Å displacement of Mn from the N_4_ plane and thus slightly longer Mn–N bonds relative to **1**. As such, to our knowledge, the structure of **1** provides the first example of a four-coordinate Mn porphyrin species. The isolation of a rigorously four-coordinate Mn center within a porphyrin ligand is remarkable given the relatively large ionic radius of Mn^II^, and thus its propensity to displace out of the N_4_ plane12*b* to form five-coordinate complexes.12*g* Indeed, the complex in **1** demonstrates the utility of MOFs to enable isolation of reactive low-coordinate metal complexes.

Exposure of single crystals of **1** to *ca.* 1 atm of dry O_2_ at –78 °C resulted in an immediate color change from purple to black. Subsequent X-ray diffraction analysis at 100 K revealed the formation of **2**. The structure of **2** is globally quite similar to that of **1**, but with key differences in the Mn coordination environment (see Fig. 3, S3, and Table S2†). Most importantly, the structure features a Mn ion that is coordinated to O_2_*via* a side-on, η^2^ binding mode. In addition, the Mn ion is displaced from the N_4_ plane by 0.80(2) Å, slightly longer than the displacement of 0.7640(4) Å observed for the peroxomanganese(iii) unit in a potassium cryptate salt of [(TPP)MnO_2_]^–^.7*a* Likewise, the Mn–N and Mn–O distances of 2.170(9) and 1.76(3) Å in **2** are slightly shorter than the analogous mean distances of 2.184(4) and 1.895(4) Å in [(TPP)MnO_2_]^–^. While these differences are consistent with the presence of a smaller ionic radius for Mn^IV^*vs.* Mn^III^, they should be regarded with caution owing to the positional disorder and large thermal ellipsoids of O atoms in **2** (see Fig. S4†). Finally, this crystallographic disorder also precludes a reliable determination of the O–O distance in **2**. Here, this distance was fixed to a target value of 1.40 ± 0.02 Å, based on other peroxometal complexes with similar values of *ν*_O–O_,^9^,^23^,^25^ and subsequent refinement of the structure gave a distance of 1.39(2) Å.

In the structure of **2**, the O_peroxo_ atoms are related through a crystallographic mirror plane. As such, the structure was modeled with an O_2_ unit bound symmetrically with respect to Mn. However, we cannot exclude the possibility of some asymmetry based on the current structure. Indeed, several previously reported crystal structures of η^2^-bound peroxometal complexes feature asymmetric coordination of the peroxo ligand.^7^,^9^ Nevertheless, the structure of **2** shows that the O–O bond eclipses the pyrrole-based N atoms. This conformation contrasts the results from charge iterative extended Hückel (IEH) calculations on a peroxomanganese(iv) porphyrin complex, which suggested the presence of a staggered peroxo ligand to alleviate electrostatic repulsion.12*j* However, the eclipsed conformation observed for **2** is consistent with those determined crystallographically for the peroxometal porphyrin complexes (OEP)TiO_2_,^23^ (TPP)Mo(O_2_)_2_,^26^ and [(TPP)MnO_2_]^–^.7*a*

Despite the large standard deviations in the structure of **2**, this analysis unambiguously reveals the presence of an O_2_ adduct coordinated to Mn through a side-on, η^2^ binding mode, with the O_2_ eclipsing the N atoms of the porphyrin pyrroles. Moreover, the complex in **2** represents a rare example of both a structurally characterized peroxometalloporphyrin7a,^23^,^26^ and a peroxomanganese(iv) complex.^9^ To our knowledge, **2** provides the first crystal structure of a peroxomanganese(iv) in a porphyrinoid ligand and a rare example of a peroxomanganese(iv) in any ligand environment.^9^,^27^

### Electron paramagnetic resonance (EPR) spectroscopy

**Fig. 4:**
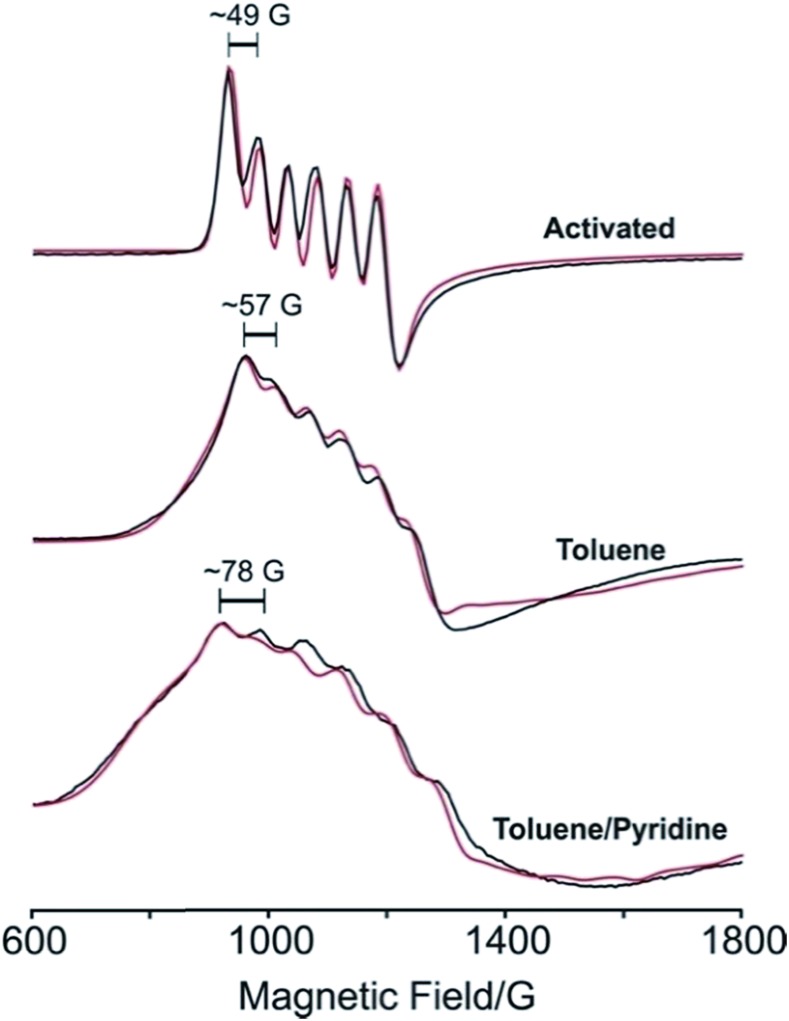
Experimental (black) and simulated (red) X-band EPR spectra of **1** at 77 K after activation (upper), in toluene (center), and in a mixture of toluene and pyridine (lower).

**Fig. 5:**
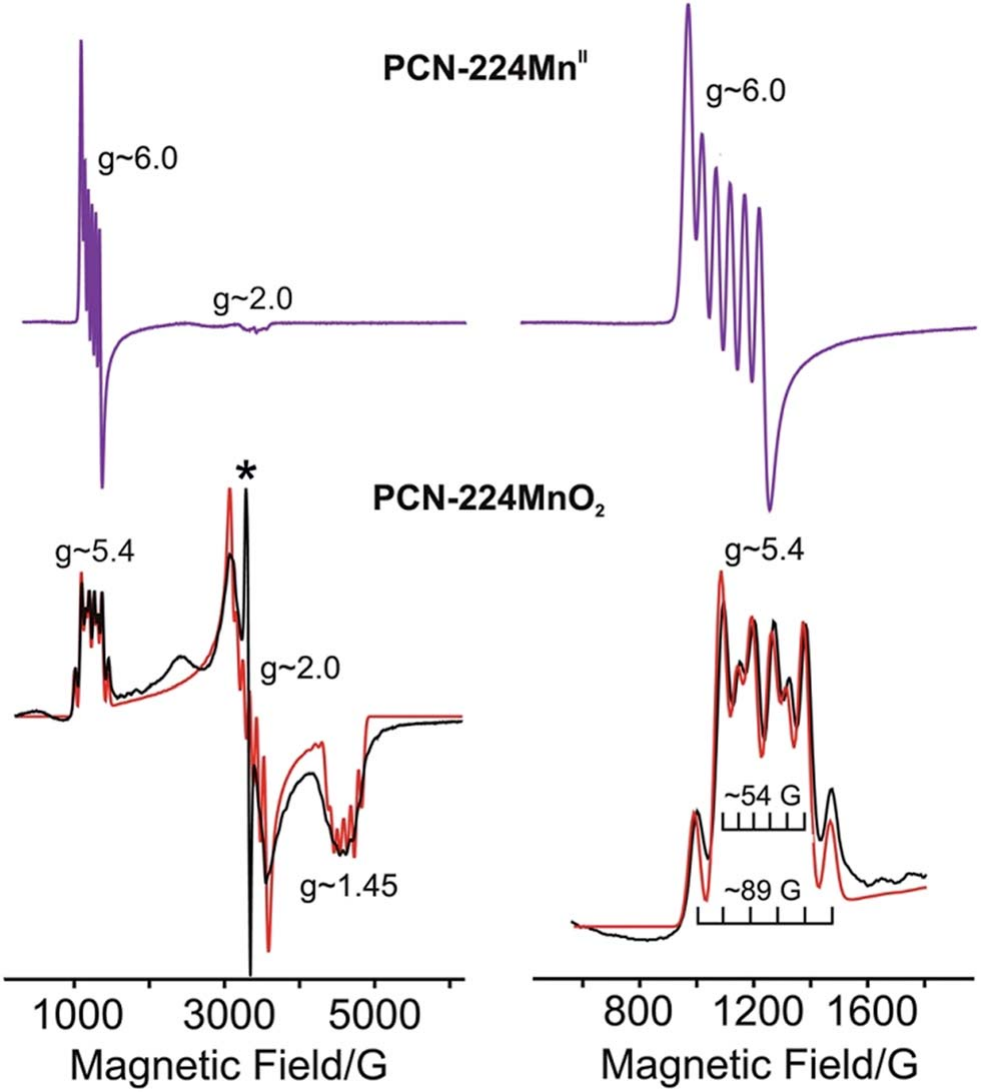
Left: X band EPR spectrum of **1** (purple lines) and **2** (black lines) at 4.2 K, with simulation shown in red. Right: Expanded view of the low-field portion of the EPR spectrum. Braces in the inset show the calculated hyperfine splittings of the superposed lower (*A*^l^ ≈ 54 G) and upper (*A*^u^ ≈ 89 G) sextets. The asterisk denotes an impurity of *S* = 1/2 with *g* = 2.00.

To further probe the electronic structures of **1** and **2**, as well as the reversibility of O_2_ binding, continuous wave X-band EPR spectra were collected on samples at selected temperatures between 298 K and 4.2 K (Fig. 4 and 5; see Fig. S5† for full temperature range). The spectrum obtained at 77 K for a rigorously activated sample of **1** shows a sharp axial pattern, with observed *g* values of *g*_⊥_ = 6 and *g*_‖_ = 2 that are characteristic of an *S* = 5/2 Mn^II^ ion with zero-field splitting parameters of *D* ≫ *hν*, *E*/*D* = *λ* = 0 (see Fig. 4, upper). In addition, the low field portion features a well-resolved six-line hyperfine splitting by the *I* = 5/2 ^55^Mn nucleus, with a hyperfine constant of *A*_⊥_^Mn^ ≈ 49 G (138 MHz).

Addition of toluene to a crystalline sample of **1** led to a broadening of the *g*_⊥_ region of the spectrum and an increase in the hyperfine coupling (Fig. 4, middle). Accordingly, simulation of the spectrum with the program Easyspin^27^ show that interaction of **1** with toluene lowers the four-fold symmetry of the Mn^II^ ion, as indicated by the introduction of a non-zero rhombicity of the zero-field splitting, *λ* ≈ 0.027, along with an increase in hyperfine coupling to *A*_⊥_^Mn^ ≈ 57 G (160 MHz). Addition of a three-fold molar excess of pyridine in toluene to **1** (Fig. 4, lower) further lowers the symmetry, as indicated by a further increase in the rhombicity and the hyperfine coupling to *λ* ≈ 0.041 and *A*_⊥_^Mn^ ≈ 78 G (219 MHz), respectively. It appears that pyridine binding introduces a rhombicity in the hyperfine interaction as well, although precisely reproducing the poorly-resolved spectrum is not illuminating. The progressive departure from four-fold symmetry is evident from increases in *λ* (see Table S3†). The change in the spectrum simply upon addition of toluene solvent strongly supports the four-coordinate nature of activated **1**, and the transformation to a five-coordinate complex even upon addition of toluene solvent, not merely with the strongly-coordinating pyridine. The progressive increase in hyperfine coupling in these three samples reflects an increasing departure of the Mn^II^ from the N_4_ plane.

Dosing **1** with *ca.* 1 atm of dry O_2_ resulted in conversion to **2**, which features a rhombic spectrum at ∼7 K (see Fig. 5, lower). As has been analysed in detail for Mn(TPP)O_2_, O_2_ binding yields a complex formally described as a high-spin Mn^IV^ peroxo species, with the rhombicity of this EPR spectrum associated with orbital mixing on the Mn^IV^.12a,c,e Following the analysis for Mn(TPP)O_2_, the spectrum represents an *S* = 3/2 spin state with axial zero-field parameter, *D* < 0 and |*D*| much greater than the microwave quantum (0.3 cm^–1^). In this case the spectrum comprises the overlap of two spectra, arising from transitions within each of the two doublets created by the zero-field splitting of the *S* = 3/2 state, and the *g*-values of those spectra depend only on the rhombicity parameter, *λ*. As with (TPP)MnO_2_, the spectrum in Fig. 4 corresponds well to the limit *λ* → 1/3, where both doublets exhibits a g-tensor with *g* values of [5.4–5.5, 2, 1.45].12*c* The feature at *g* = 5.4 exhibits the overlap of two ^55^Mn hyperfine sextet patterns, where *A*^l^ = 54 G (154 MHz) for the lower doublet and *A*^u^ = 89 G (251 MHz) for the upper doublet (see Fig. 5 and Table S3†).12a,c Orbital mixing on Mn^IV^ causes the difference in the observed ^55^Mn hyperfine splitting for the two doublets.^18^,^28^

The ratio of signal intensities for the two doublets is determined by the Boltzmann populations of the two doublets at the measurement temperature, as determined by the energy difference between them, *Δ* = 2(*D*^2^ + 3*E*^2^)^1/2^ = 2|*D*|(1 + 3*λ*^2^)^1/2^. Taking into account the negative sign of *D*, the simulated overlapping spectra and their relative intensities at *T* 4.2 K are nicely reproduced using the measured hyperfine coupling and, *D* = –1.76 cm^–1^, *λ* = 1/3, with a corresponding zero-field energy separation of the two doublets, *Δ* = 4.07 cm^–1^. These values of *D*, *Δ* can be compared to *D* = –2.48 cm^–1^, *λ* ∼1/3, *Δ* = 5.82 cm^–1^ for Mn(TPP)O_2_.

As the temperature is increased from 4.2 K, the populations of the two doublets begin to equalize and the spectra broaden, likely in some part because of rotation of the O_2_ (see Fig. S5†). Finally, when **2** was purged with Ar at 298 K and evacuated for 12 h, a spectrum identical to that for **1** was obtained (see Fig. S6†). This observation corroborates the reversibility of O_2_ binding at Mn as revealed by DRIFTS analysis.

### O_2_ adsorption

**Fig. 6:**
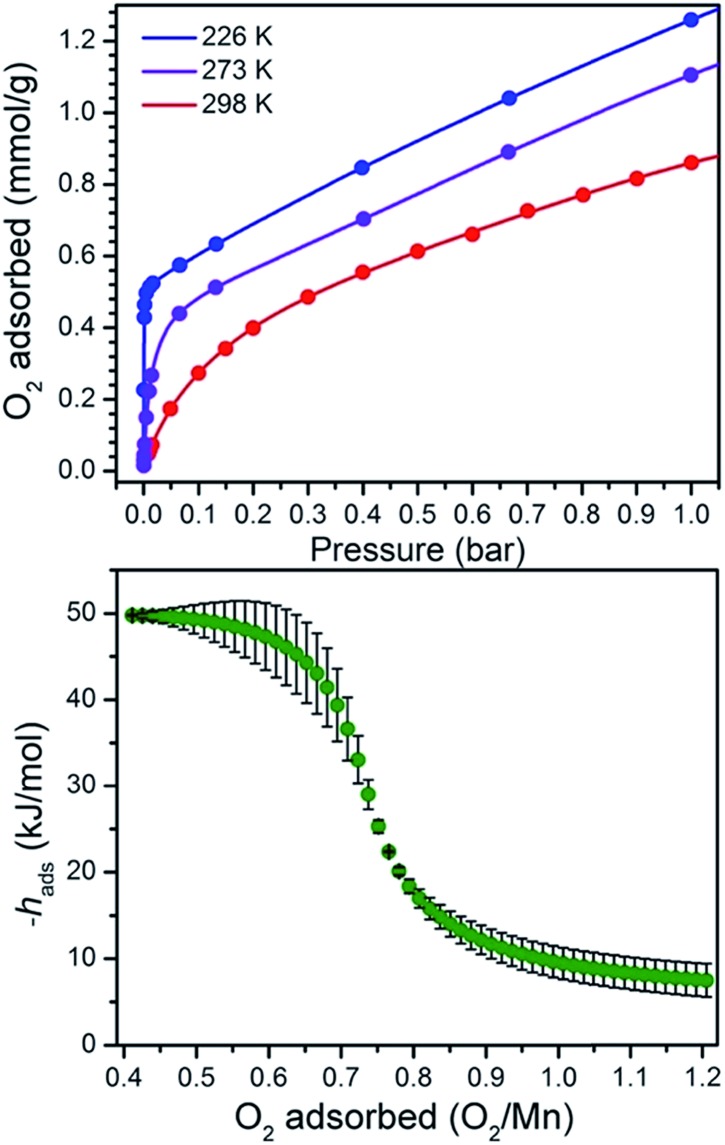
Upper: O_2_ adsorption data for **1** at 233, 273, and 298 K (blue to red gradient). Circles represent data, and solid lines correspond to fits using a dual-site Langmuir–Freundlich model. Lower: O_2_ differential enthalpy of adsorption curve for **1**, plotted as a function of O_2_ adsorbed. Green circles represent data, and error bars are shown in black.

The reversibility of the O_2_ interaction enables quantitation of the O_2_ binding thermodynamics in PCN-224Mn^II^ through variable temperature O_2_ adsorption measurements. Toward this end, O_2_ uptake data were collected at selected temperatures between 233 and 298 K, as depicted in Fig. 6 and S7–S8. At 233 K, the O_2_ isotherm exhibits an initial steep uptake at low pressure. As the temperature is increased, the slope of this steep region decreases until the isotherm becomes closer to linear at 298 K. To quantify the strength of O_2_ binding, the isotherm data were fit to a dual-site Langmuir–Freundlich model (see Table S4†), and subsequent treatment of the variable-temperature data with the Clausius–Clapeyron equation revealed a differential enthalpy of adsorption of *h*_ads_ = –49.6(8) kJ mol^–1^ at low loading, followed by a gradual drop near 1 : 1 Mn : O_2_ to a plateau at *h*_ads_ = –9(2) kJ mol^–1^. We assign these values to O_2_ binding at the Mn center and physisorption to the remainder of the MOF surface, respectively.

The binding of O_2_ to Mn^II^ in PCN-224Mn^II^ is significantly stronger than the analogous values measured for O_2_ binding at the four-coordinate metal centers in PCN-224Fe^II^ and PCN-224Co^II^ of –34(4) and –15.2(6) kJ mol^–1^, respectively.^16^,^18^ As previously discussed, the difference in binding enthalpy can primarily be attributed to the difference in the redox couple at the metal center, which is associated with electron transfer upon the binding of O_2_.12a,^29^ In line with this relationship, the enthalpy of M–O_2_ binding from Mn to Fe to Co in PCN-224M^II^ decreases as *E*_1/2_ (*V vs.* SCE) of M^II/III^ of the analogous molecular metalloporphyins becomes less reducing (Mn: –0.230; Fe: –0.047; Co: +0.320).^29^ Note, however, that the binding enthalpy in Mn is further strengthened by the fact that O_2_ binding involves a two-electron transfer from Mn to O_2_.

## Conclusions

The foregoing results demonstrate the ability of a MOF to enable the isolation and crystallographic characterization of a four-coordinate manganese porphyrin center and its corresponding O_2_ adduct. A combined array of single-crystal X-ray diffraction, solid-state infrared, and EPR analysis collectively demonstrate the O_2_ complex to comprise a peroxo ligand bound in a side-on, η^2^ mode to an *S* = 3/2 Mn^IV^ ion. In addition, these experiments reveal that O_2_ binding is reversible, even at ambient temperature, in stark contrast to behaviour observed in molecular analogues. Finally, O_2_ gas adsorption measurements quantify the enthalpy of O_2_ binding as *h*_ads_ = –49.6(8) kJ mol^–1^. This value is considerably higher than in the corresponding Fe- and Co-based MOFs, and the strength of binding is found to increase with increasing reductive capacity of the M^II/III^ redox couple. Work is underway to carry out and study the protonation, O–O bond cleavage, and O-atom transfer ability of the peroxo ligand, with emphasis on isolating and structurally characterizing the intermediates involved in these processes.

## Conflicts of interest

There are no conflicts to declare.

## Supplementary Material

Supplementary information

Crystal structure data
