# Supplementary material for: A structurally-characterized peroxomanganese(iv) porphyrin from reversible O2 binding within a metal–organic framework
Source: Chem Sci. 2017 Dec 14;9(6):1596–603. doi: 10.1039/c7sc03739b (PMC5890324; doi:10.1039/c7sc03739b)
Supplement: Supplementary file 1 [file SC-009-C7SC03739B-s001.pdf]

Electronic Supplementary Material (ESI) for Chemical Science.
This journal is © The Royal Society of Chemistry 2017

Electronic Supplementary Material for:

A Structurally-Characterized Peroxomaganese(IV) Porphyrin from Reversible O_2 Binding within a Metal-Organic Framework

Audrey T. Gallagher, Jung Yoon Lee, Venkatesan Kathiresan, John S. Anderson,[a] Brian M. Hoffman, and T. David Harris*

Department of Chemistry, Northwestern University, 2145 Sheridan Road, Evanston, Illinois 60208-3113, USA
[a]*Current address: Department of Chemistry, University of Chicago, 929 E. 57th Street, Chicago, Illinois 60637-1454, USA*

Email:
T. D. Harris, dharris@northwestern.edu

Chem. Sci.

Table of Contents

Experimental Section **S3**
Figure S1: Powder X-ray Diffraction of PCN-224Mn^{II} **S6**
Figure S2: N_2 adsorption data for PCN-224Mn^{II} **S7**
Figure S3: IR spectra for PCN-224Mn^{II} before and after the addition of O_2 **S8**
Figure S4: Thermal ellipsoid plots for PCN-224Mn^{II} and PCN-224MnO_2 **S9**
Table S1: Crystallographic table for PCN-224Mn^{II} **S10**
Table S2: Crystallographic table for PCN-224MnO_2 **S11**
Figure S5: Variable temperature EPR spectra for PCN-224MnO_2 **S12**
Table S3: Selected spin-Hamiltonian parameters obtained from EPR simulations **S13**
Figure S6: EPR spectra demonstrating the reversibility of O_2 binding **S14**
Figure S7: Comparison of O_2 adsorption data for PCN-224 and PCN-224Mn^{II} **S15**
Figure S8: O_2 adsorption and desorption data for PCN-224Mn^{II} **S16**
Table S4: Langmuir fit parameters for PCN-224Mn^{II} plus O_2 **S17**
Figure S9: Characteristic X-ray diffraction pattern of PCN-224Mn^{II} **S18**
References **S19**

Experimental Section

General Considerations. Unless otherwise noted, all materials and chemicals were purchased from commercial suppliers and used without further purification. Additionally, unless otherwise stated, all manipulations were carried out under an atmosphere of dinitrogen using either standard Schlenk techniques or in a Vacuum Atmospheres Nexus II glovebox. All glassware was dried at 150 °C and allowed to cool under vacuum prior to use. All solvents were dried on a solvent purification system from Pure Process Technology and stored under N_2 over 4 Å molecular sieves. Effective removal of O_2 and H_2O from solvents was verified using a standard solution of Na benzophenone ketyl radical anion. The material PCN-224 was prepared as previously reported.[1,2]

Synthesis of PCN-224Mn^{II} (1). A 20 mL vial was charged with PCN-224 (100 mg, 0.0248 mmol), $MnBr_2$ (50 mg, 0.233 mmol), DMF (5 mL), and 2,6-lutidine (1 g). This vial was then sealed and heated for 12 h at 150 °C. After this time, the supernatant was decanted off and the remaining solid was washed with DMF (10 × 5 mL) by soaking the material for 30 min at 150 °C and decanting. The solid was then washed with THF (10 × 5 mL) followed by MeOH (10 × 5 mL) by soaking the material for 1 h and then decanting off the supernatant. The remaining solid was then dried for 12 h at 150 °C under vacuum to yield **1** as a dark purple crystalline solid (0.098 g, 93%). Note that all subsequent manipulations of this compound were carried out in a solvent-free atmosphere glovebox. The experimental BET surface area of 2455 m^2/g is close to the accessible surface area reported for other metalated variants of PCN-224, which confirms material porosity and successful removal of solvent from the pores (see Figure S1).[1,2,3,4] Complete metalation was confirmed by ICP-AES (Zr:Mn mass ratio: expected 6.6; found 7.0) and UV/Visible spectroscopy (see Figure 1).

Synthesis of PCN-224MnO$_2$ (2). For bulk measurements, this compound was generated by exposing freshly-activated **1** (100 mg, 0.0229 mmol) to ca. 1 atm of dry O_2. Samples for single-crystal X-ray diffraction were prepared by cooling activated crystals of **1** to −78 °C and then introducing 1 atm of O_2 for 10 min on a Schlenk line. After this time, the crystals were coated in Paratone-N oil with a backflow of O_2, transported while cold, and rapidly mounted on the diffractometer.

Diffuse-Reflectance UV/Visible Spectroscopy. The sample was prepared as a 10-fold dilution of **1** in KBr and pulverized with a mortar and pestle to make a smooth, homogenous powder. The samples were then transferred to a Harrick Praying Mantis™ reaction chamber under a N_2 atmosphere. Solid-state UV/Visible spectra were obtained using an Agilent Cary 5000 spectrophotometer at room temperature. The data were treated with a background correction of KBr and the spectra are reported as Kubelka-Munk transform.

Solution UV/Visible Spectroscopy. A sample of **1** was pulverized with a mortar and pestle and transferred as a toluene suspension to a 3 mL (1 cm pathlength) quartz UV/VIS cuvette under a N_2 atmosphere. UV/Visible spectra were obtained using an Agilent Cary 5000 spectrophotometer

at room temperature. A baseline of toluene was taken prior to the measurement of the sample of interest.

Diffuse-Reflectance Infrared Fourier Transform Spectroscopy (DRIFTS). The sample was prepared as a 50-fold dilution of **1** in KBr and pulverized with a mortar and pestle to make a smooth, homogenous powder. The sample was then transferred to a Harrick Praying Mantis™ low-temperature reaction chamber equipped with a gas inlet and outlet, allowing Ar and O_2 to flow through the sample. Data collections were performed on a Thermo Nicolet 6700 FT-IR spectrometer operating at a resolution of 4 cm^{-1} at 298 K. Compound **1** was purged at 298 K for 20 min with Ar and then exposed to an O_2/N_2 gas mixture for 20 min. Three iterative Ar and O_2/N_2 cycles were performed to demonstrate the reversibility of the oxygenation (see Figure S2). The collected data were treated with a background correction of the reaction chamber without a sample. Data were collected at the Northwestern University Clean Catalysis (CleanCat) Core Facility.

X-ray Structure Determination. Single crystals of **1** and **2** suitable for X-ray analysis were coated in Paratone-N oil and mounted on a Micro Mounts™ rod attached to a goniometer head. The crystallographic data were collected at 100 K on a Bruker APEX II diffractometer equipped with CuKα microsource. Raw data were integrated and corrected for Lorentz and polarization effects using Bruker APEX2 v. 2009.1.4 Absorption corrections were applied using SADABS.[5] Space group assignments were determined by examination of systematic absences, E-statistics, and successive refinement of the structures. Structures were solved and refined with SHELXL[6] operated within the Olex2 interface with the aid of standard restraints.[7,8]

Disorder in **1** and **2** was modeled by splitting the atomic coordinates, and residual electron density found in the difference Fourier map was removed using the solvent mask protocol included in Olex2. This residual electron density likely arises from partial occupation of a separate morphology of the closely related MOF-525.[9] Due to the disorder of the O_2 adduct in **2**, the O1–O1 and O2–O2 bond distances were restrained to refine to expected O–O bond distances of other previously characterized transition metal peroxo complexes.[10,11,12] Specifically, this distance was fixed to a target value of 1.40 ± 0.02 Å using the soft restraint DFIX, and subsequent refinement gave an O–O distance of 1.39(2) Å. The Mn–O1 and Mn–O2 as well as the Mn–N3 distances were also constrained to equal each other to ensure physical correctness of the system. The values reported in the manuscript are the mean distances obtained from these bonds. Note that the atoms comprising the metalloporphyrin unit are elongated out of the plane of the porphyrin due to the intrinsic disorder of the system. See Figure S7 for a characteristic diffraction image of PCN-224 that highlights the diffuse scattering of the Bragg peaks due to the inherent disorder of the system and gives rise to the elongated thermal parameters for some of the atoms. See Figure S3 for thermal ellipsoid plots and Tables S1 and S2 for experimental crystallographic information.

Electron Paramagnetic Resonance (EPR) Spectroscopy. An activated sample of PCN-224Mn^{II} was loaded into a quartz EPR tube under a N_2 atmosphere and capped with a septum. The quartz EPR tube was then evacuated on a Schlenk line for 12 h at 150 °C, an EPR spectra was then collected on the evacuated sample revealing the formation of **1**. The same sample was then dosed with ca. 1 atm of dry O_2 at ambient temperature with the EPR spectrum revealing the formation of **2**. The same sample was then purged with Ar and evacuated with a corresponding

EPR spectrum revealing the restoration of the deoxygenated manganese(II) porphyrin center, **1**. Continuous-wave EPR spectra were collected on **1** at 77 K and **2** at 298, 77, 20, and 4.2 K. An activated sample of **1** (30 mg, 0.000749 mmol) was loaded into a Vac Suprasil® EPR tube equipped with a J Young valve under a N_2 atmosphere followed by the addition of toluene (1 mL) to yield PCN-224Mn^{II}•(C_7H_8). To the same sample, a solution of pyridine (5.2 mg, 0.066 mmol) dissolved in toluene (0.50 mL) was added to yield PCN-224Mn^{II}•(C_5H_5N)**.** Continuous-wave EPR spectra were collected on these two samples at 77 K. Measurements were performed using a Bruker ESP 300 X-band EPR spectrometer. Temperatures were held constant using an Oxford Instruments ESR 900 continuous-flow helium cryostat. The spectrometer was equipped with a dual mode cavity, operating in perpendicular mode. Data were collected using the following instrumental parameters: radiation frequency = 9.3684 GHz; microwave power = 2 mW; modulation amplitude = 13 G; modulation frequency = 100 kHz. Spectral simulations were carried out using the program Easyspin 5.0.16.[13]

Gas Adsorption Measurements. Crystalline samples of PCN-224 or **1** were transferred into a pre-weighed analysis tube, which was then sealed with a Micromeritics TranSeal™. Activation and analysis was then performed on a Micromeritics ASAP 2020 instrument. The samples were activated at 150 °C until an outgas rate of less than 1 mTorr/minute was observed. After activation, the samples were weighed to determine the final mass of analyte. The sample was checked to ensure the outgas rate remained below 1 mTorr/minute. O_2 uptake was measured using volumetric methods and the free space of all samples was determined with UHP He prior to analysis. The analysis was performed on both PCN-224 and **1** to demonstrate that the manganese center is responsible for the strong, initial uptake of O_2 (see Figure S6). Temperature control was provided with a variety of cold baths: dry ice/acetonitrile for 226 K, ice bath for 273 K, and no bath was used for the 298 K data collection.

Differential Enthalpies of Adsorption Calculations. The variable-temperature O_2 adsorption isotherms at 226, 273 and 298 K were independently fit with a dual-site Langmuir-Freundlich model (Eqn 1), where n is the amount adsorbed in mmol/g, P is the pressure in bar, $n_{\mathrm{sat,i}}$ is the saturation capacity in mmol/g, v_{i} is the Freundlich parameter, and b_i is the Langmuir parameter in bar^{-v} for two sites 1 and 2.

$$n = \frac{n_{\mathrm{sat},1} b_1 P^{v_1}}{1 + b_1 P^{v_1}} + \frac{n_{\mathrm{sat},2} b_2 P^{v_2}}{1 + b_2 P^{v_2}} \qquad (1)$$

Note that the Langmuir-Freundlich model was simply used to mathematically fit each adsorption isotherm in preparation for isosteric heat of adsorption calculations using the Clausius-Clapeyron equation, and we do not intend to attribute any physical meaning to the obtained parameters. The fitted parameters for each adsorption isotherm can be found in Table S4.

The Clausius-Clapeyron equation (Eqn 2) was used to calculate the differential enthalpies of adsorption, $-h_{\mathrm{ads}}$, using the dual-site Langmuir-Freundlich fits at each temperature. Here, P is the pressure, n is the amount of gas adsorbed, T is the temperature, R is the universal gas constant, and C is a constant.

$$\ln P = \frac{h_{\mathrm{ads}}}{R}\left(\frac{1}{T}\right) + C \qquad (2)$$

The isosteric heats of adsorption were obtained from the slope of plots of $(\ln P)_{\mathrm{n}}$ versus $1/T$. An error in the isosteric heat for a given loading can be calculated from the standard error in the

slope of the best-fit line. Fundamentally, this error describes the quality of agreement between the fitted isotherms and the Clausius-Clapeyron relation.

Other Physical Measurements. Inductively coupled plasma atomic emission (ICP-AE) spectra were collected with a Thermo iCAP 7600 ICP-OES instrument.

Powder X-ray Diffraction (PXRD) Analysis. An activated powder sample of PCN-224Mn^{II} under a N_2 atmosphere was loaded into two 0.7 mm boron-rich X-ray capillaries (Charles-Supper Company) and sealed with Kapton tape. PXRD data were collected at room temperature on a STOE-STADIMP powder diffractometer equipped with an asymmetric curved Germanium monochromator (Cu Kα1 radiation, λ = 1.54056 Å). The samples were measured in a transmission geometry utilizing a rotating capillary holder. See Figure S6 for the diffractogram of PCN-224Mn^{II}.

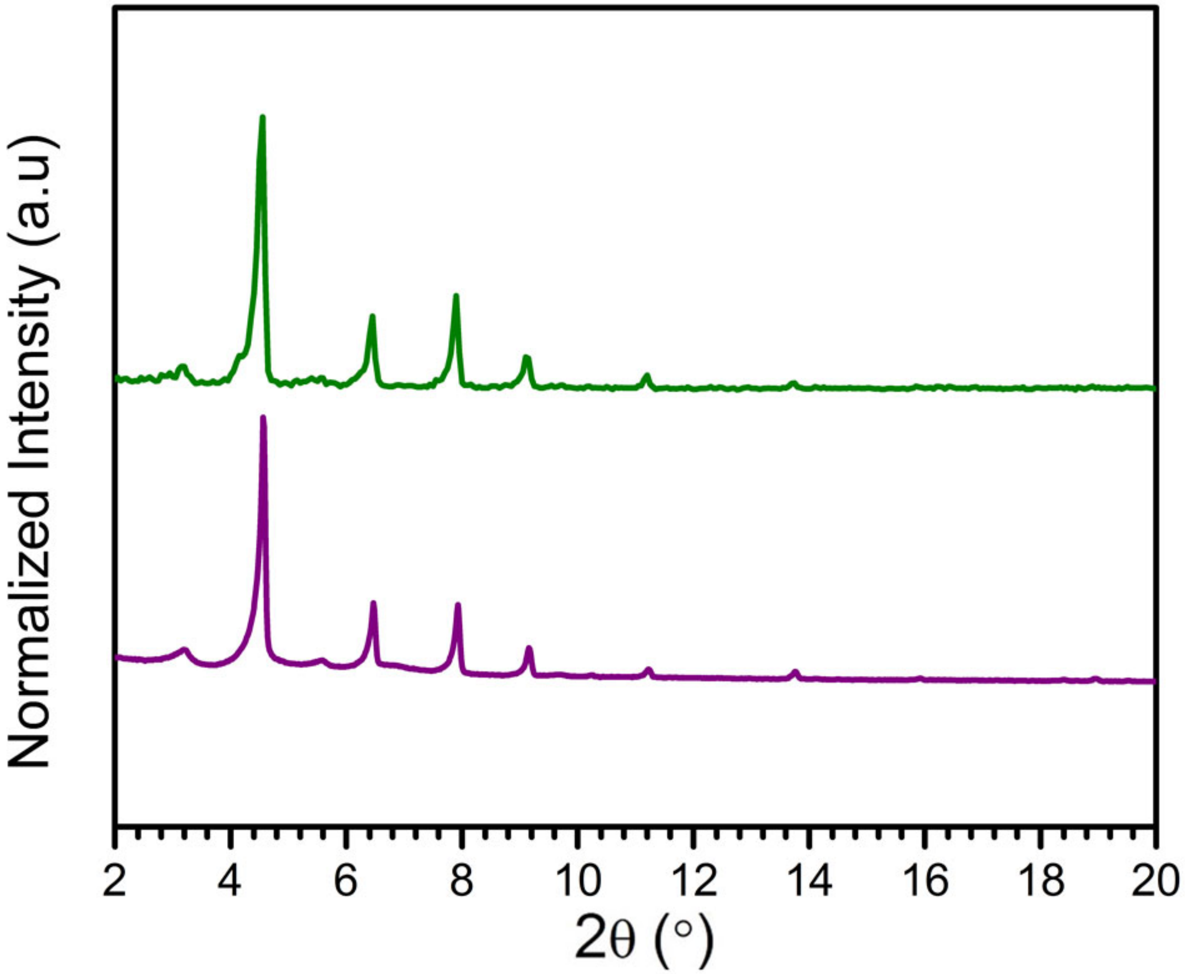

Figure S1 | Powder X-ray diffraction analysis of PCN-224 (green) and PCN-224Mn^{II} (purple) demonstrating the retention of bulk-phase purity after metalation.

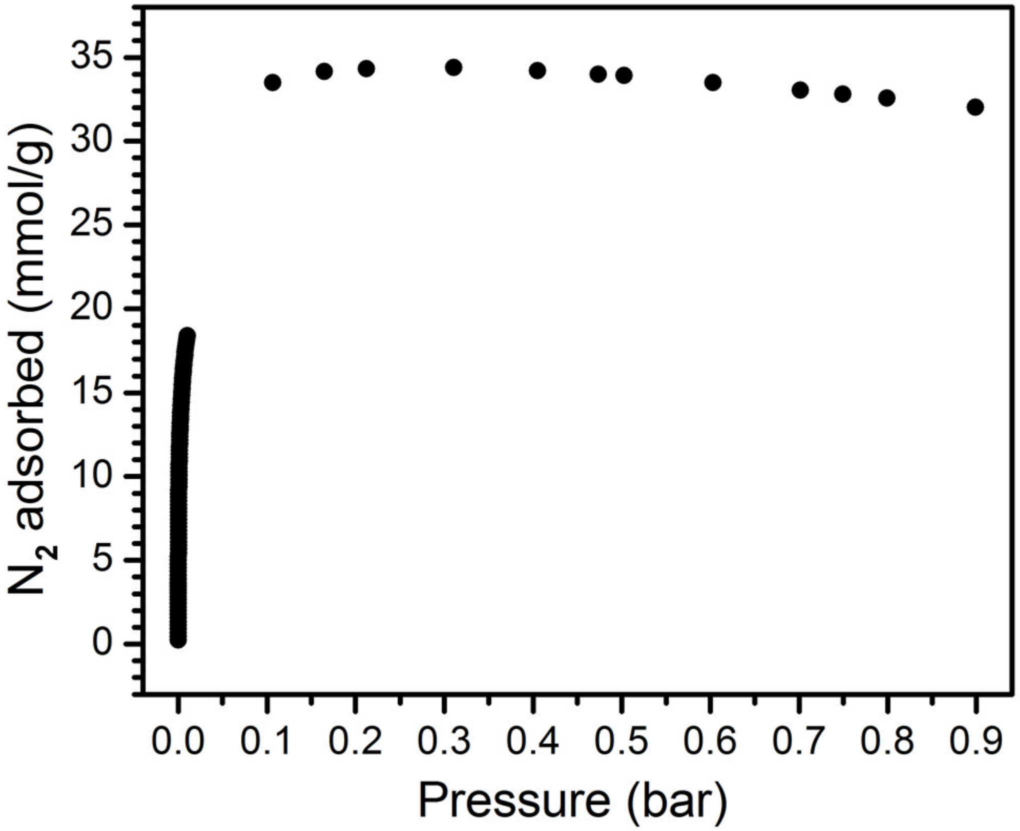

Figure S2 | N_2 adsorption data for **1** at 77 K.

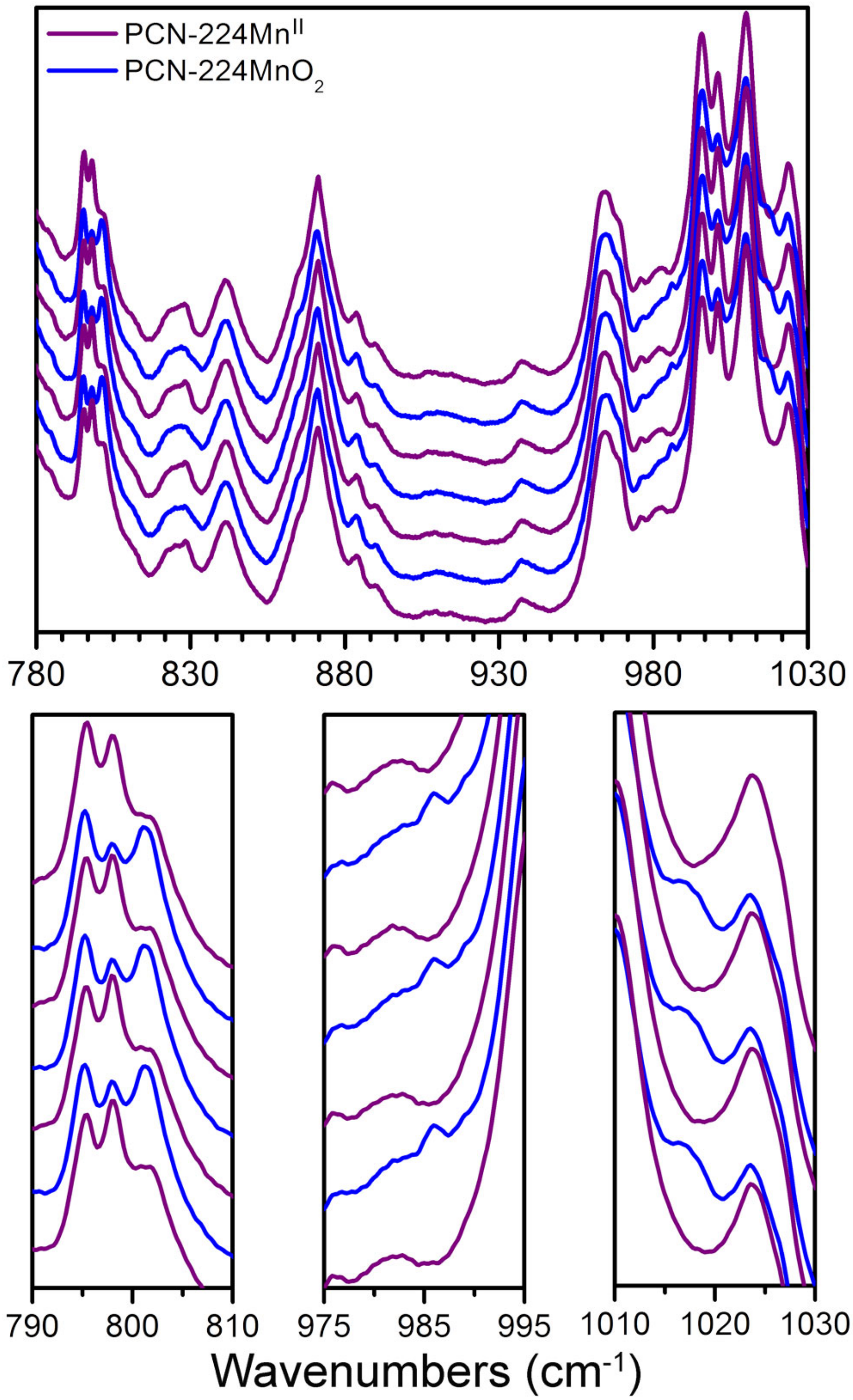

Figure S3 | Diffuse-reflectance infrared Fourier transform spectra for a desolvated sample **1** at 298 K after iteratively purging with Ar (purple) and O_2 (blue), highlighting the reversibility of O_2 binding. The bottom three panels highlight the regions that noticeably change upon the addition of O_2, with new vibrations that appear in the blue spectra at 801 cm^{-1} (bottom left), 984 cm^{-1} (bottom middle), and 1017 cm^{-1} (bottom right).

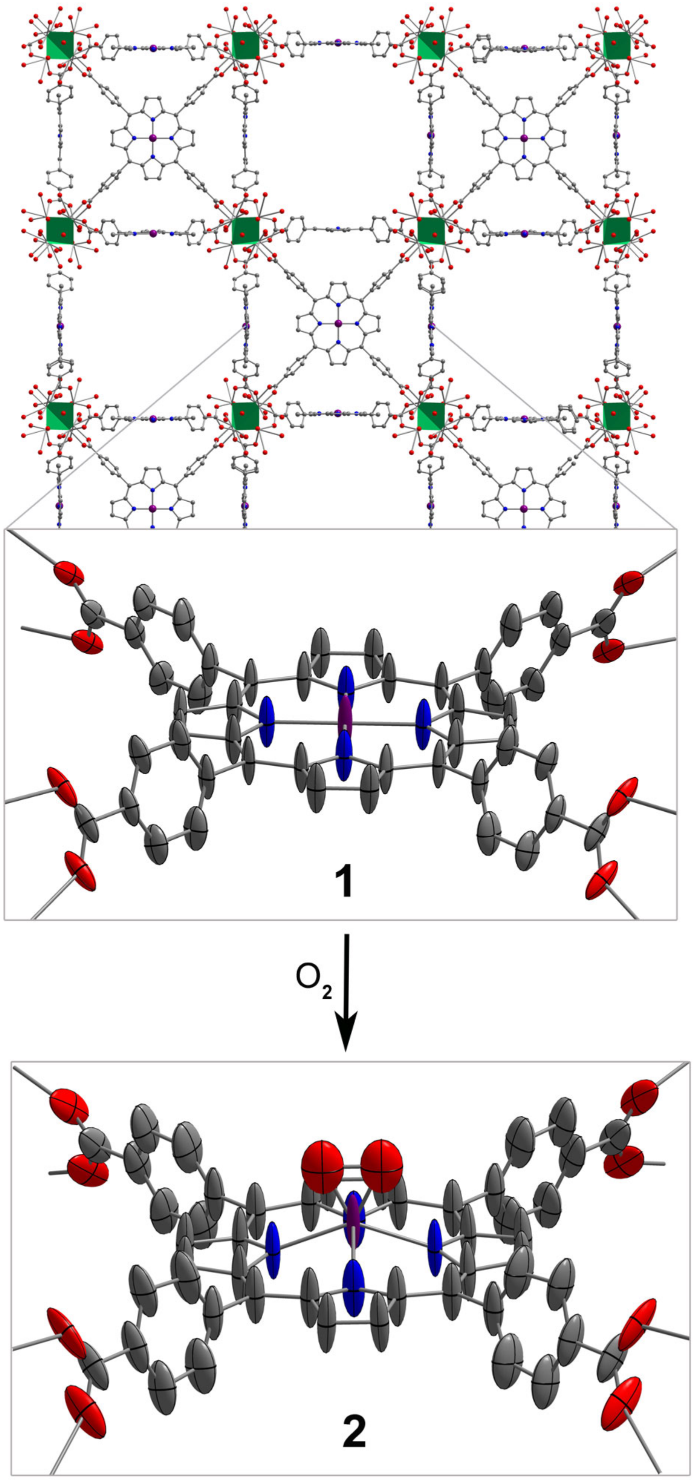

Figure S4 | Reaction of PCN-224Mn^{II} (**1**) with O_2 at −78 ˚C to form PCN-224MnO_2 (**2**). Ellipsoids shown at 30% probability level. Vertices of the green octahedra are Zr atoms; purple, blue, red, and gray spheres represent Mn, N, O, and C atoms, respectively; H atoms are omitted for clarity.

Table S1 | Crystal data and structure refinement for **1** at 100 K.

Empirical formula	$C_{144}H_{72}Mn_3N_{12}O_{52}Zr_{12}$
Formula weight	4061.59
Temperature	100.0 K
Wavelength	1.54178 Å
Crystal system	cubic
Space group	I m -3 m
Unit cell dimensions	a = 38.6720(10) Å, α = 90°<br>b = 38.6720(10) Å, β = 90°<br>c = 38.6720(10) Å, γ = 90°
Volume	57835(4) $Å^3$
Z	4
Density (calculated)	0.466 g/cm^3
Absorption coefficient	2.409 mm^{-1}
F(000)	7964
Crystal size	0.204 x 0.2 x 0.15 mm^3
θ range for data collection	2.285 to 68.349°
Index ranges	-46<=h<=44, -42<=k<=46, -45<=l<=44
Reflections collected	154240
Independent reflections	4950 [R_{int} = 0.0934]
Completeness to θ = 67.679°	100%
Refinement method	Full-matrix least-squares on F^2
Data / restraints / parameters	4950 / 211 / 102
Goodness-of-fit	1.034
Final R indices [I > 2σ(I)]	R_{obs} = 0.0844, wR_{obs} = 0.2432
R indices [all data]	R_{all} = 0.0949, wR_{all} = 0.2639
Largest diff. peak and hole	1.489 and -0.737 $e\cdot Å^{-3}$

$R = \Sigma||F_o|-|F_c|| / \Sigma|F_o|$, $wR = \{\Sigma[w(|F_o|^2 - |F_c|^2)^2] / \Sigma[w(|F_o|^4)]\}^{1/2}$ and $w = 1/[\sigma^2(Fo^2)+(0.1790P)^2+83.5939P]$ where $P = (Fo^2+2Fc^2)/3\sigma^2(I)+0.0004I^2)$

Table S2 | Crystal data and structure refinement for **2** at 100 K.

Empirical formula	$C_{144}H_{72}Mn_3N_{12}O_{76}Zr_{12}$
Formula weight	4445.24
Temperature	100 K
Wavelength	1.54178 Å
Crystal system	cubic
Space group	I m -3 m
Unit cell dimensions	a = 38.4015(12) Å, α = 90°<br>b = 38.4015(12) Å, β = 90°<br>c = 38.4015(12) Å, γ = 90°
Volume	56630(5) $Å^3$
Z	4.00032
Density (calculated)	0.521 g/cm^3
Absorption coefficient	2.512 mm^{-1}
F(000)	8732
Crystal size	0.213 x 0.196 x 0.156 mm^3
θ range for data collection	1.627 to 68.292°
Index ranges	-46<=h<=46, -44<=k<=46, -37<=l<=44
Reflections collected	148036
Independent reflections	4843 [R_{int} = 0.0723]
Completeness to θ = 67.679°	100%
Refinement method	Full-matrix least-squares on F^2
Data / restraints / parameters	4843 / 232 / 121
Goodness-of-fit	2.020
Final R indices [I > 2σ(I)]	R_{obs} = 0.1458, wR_{obs} = 0.4135
R indices [all data]	R_{all} = 0.1580, wR_{all} = 0.4338
Largest diff. peak and hole	2.556 and -1.034 e·$Å^{-3}$

$R = \Sigma||F_o|-|F_c|| / \Sigma|F_o|$, $wR = \{\Sigma[w(|F_o|^2 - |F_c|^2)^2] / \Sigma[w(|F_o|^4)]\}^{1/2}$ and $w = 1/[\sigma^2(Fo^2)+(0.2000P)^2]$ where $P = (Fo^2+2Fc^2)/3$

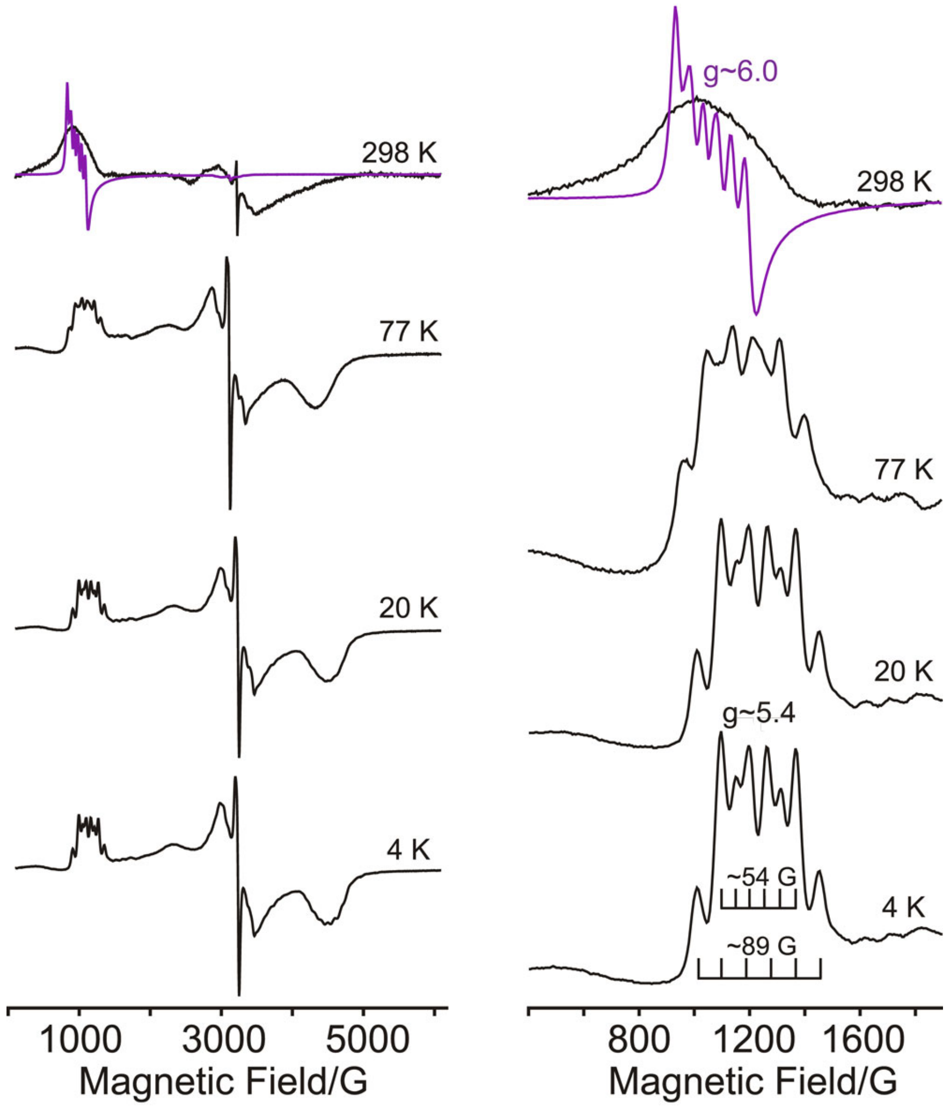

Figure S5 | EPR spectra **2 (**black) at various temperatures with the corresponding spectra of **1** (purple) highlighting changes to the electronic structure of the Mn ion upon the addition of O_2. Selected EPR simulation parameters for **1** and **2** are provided adjacent to the spectra.

Table S3 | Selected spin-Hamiltonian parameters obtained from EPR simulations.

Compound	D (cm^{-1})	λ	A (cm^{-1} × 10^{4})
PCN-224Mn^{II} **(1)**	0.55	0	46.032
PCN-224Mn^{II} + C_7H_8	0.55	0.0267	53.579
PCN-224Mn^{II} + C_5H_5N	0.55	0.0410	73.385
PCN-224Mn$^{IV}O_2$ **(2)**	−1.76	$^{1}/_{3}$	50.759, 83.659

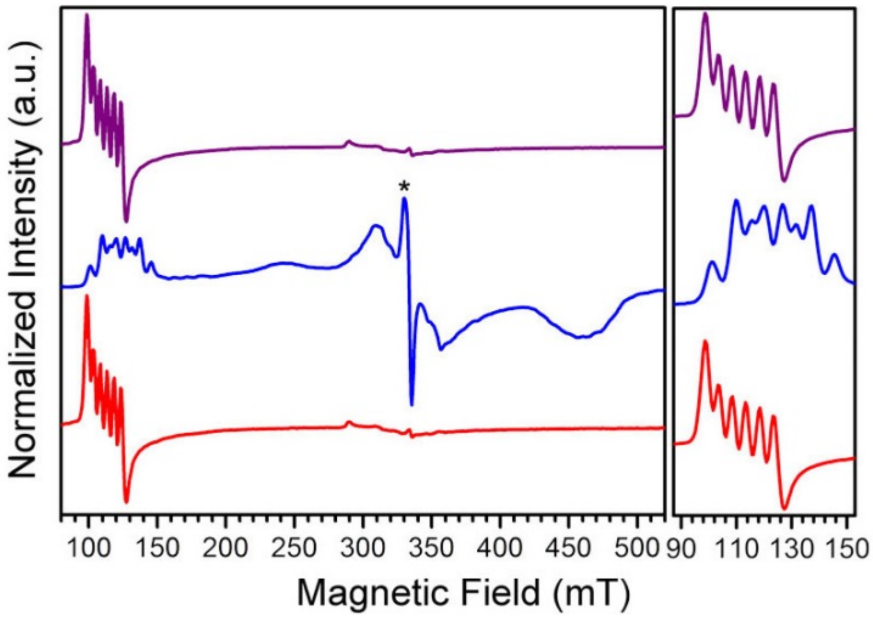

Figure S6 | X-band EPR spectra collected at 4.2 K for **1**, under static vacuum (purple), dosed with 1 atm O_2 at ambient temperature to form **2** (blue), and an evacuated sample of **2** showing conversion back to **1** (red) due to reversible O_2 binding. Asterisk denotes an impurity of $S = 1/2$ with g = 2.00. Full spectrum is shown on the left and zoomed in region highlighting the ^{55}Mn hyperfine coupling on the right.

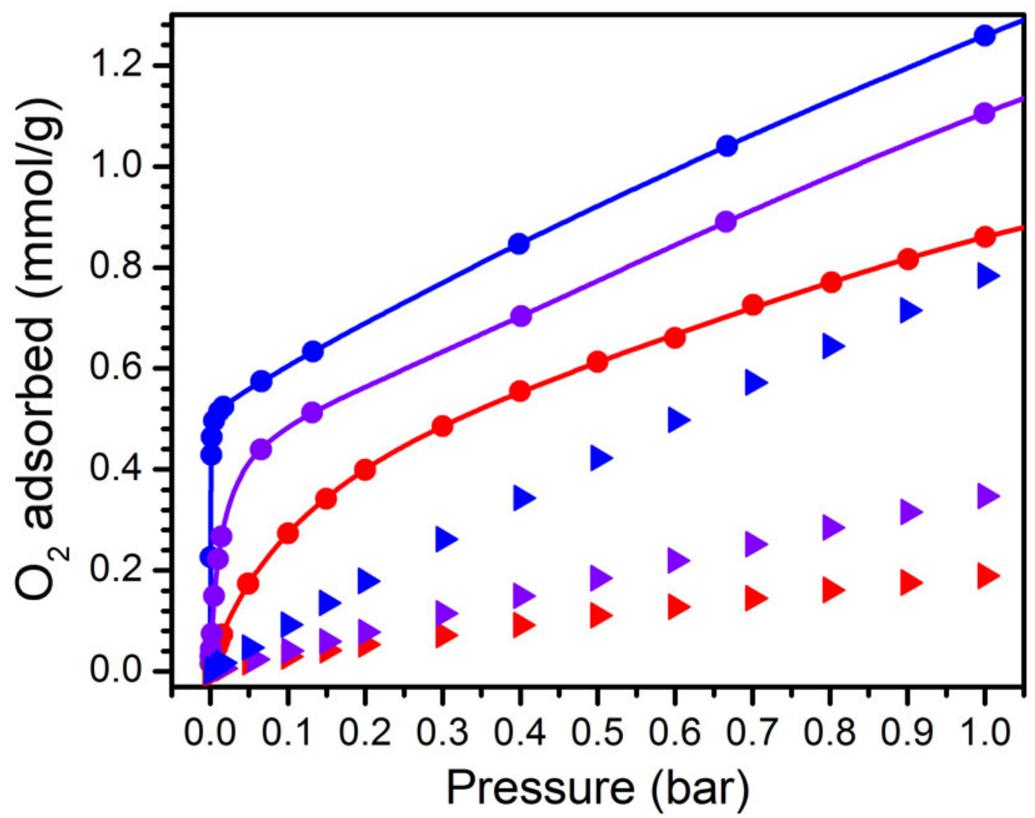

Figure S7 | O_2 adsorption data for PCN-224 (triangles) and **1** (circles) at 226, 273, and 298 K (blue to red gradient). Solid lines correspond to fits using a dual-site Langmuir-Freundlich model.

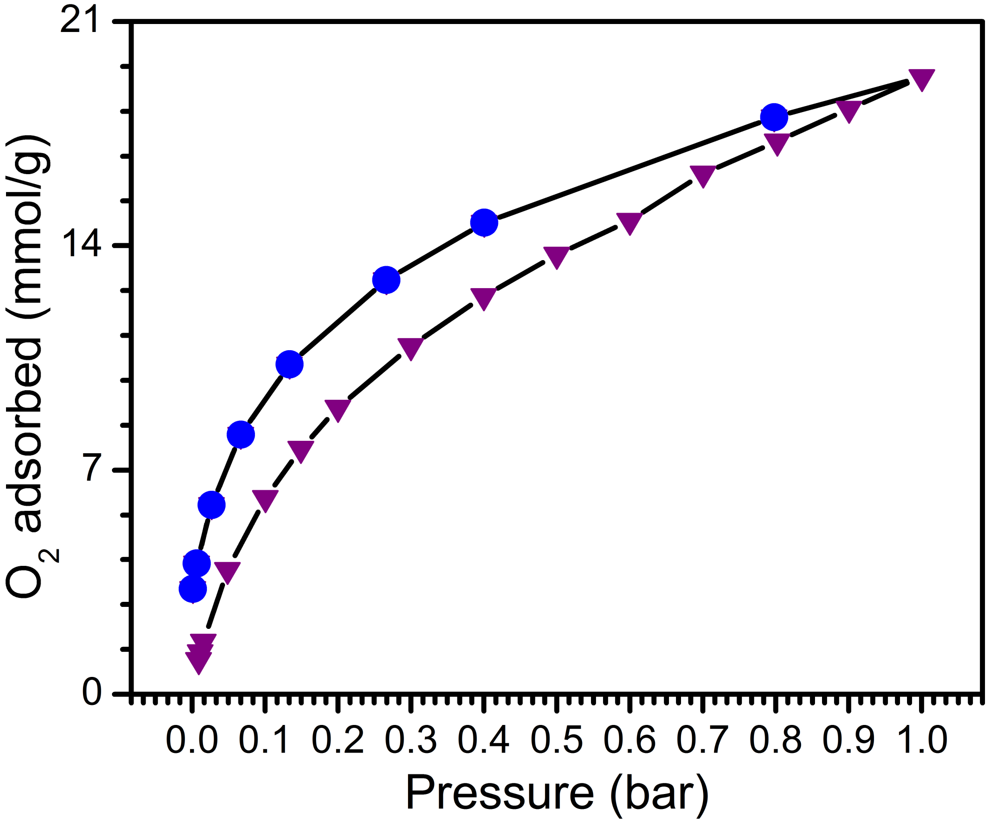

Figure S8 | O_2 adsorption data (purple triangles) and desportion data (blue circles) **1** at 298 K. (

Table S4 | Dual-site Langmuir-Freundlich fit parameters for O_2 adsorption.

	233 K	273 K	298 K
$n_{sat,1}$	6.09	50	50
b_1	0.139	0.0107	0.011
v_1	0.955	0.778	0.778
$n_{sat,2}$	0.512	0.363	0.363
b_2	10078.856	10	10
v_2	1.146	0.980	0.980

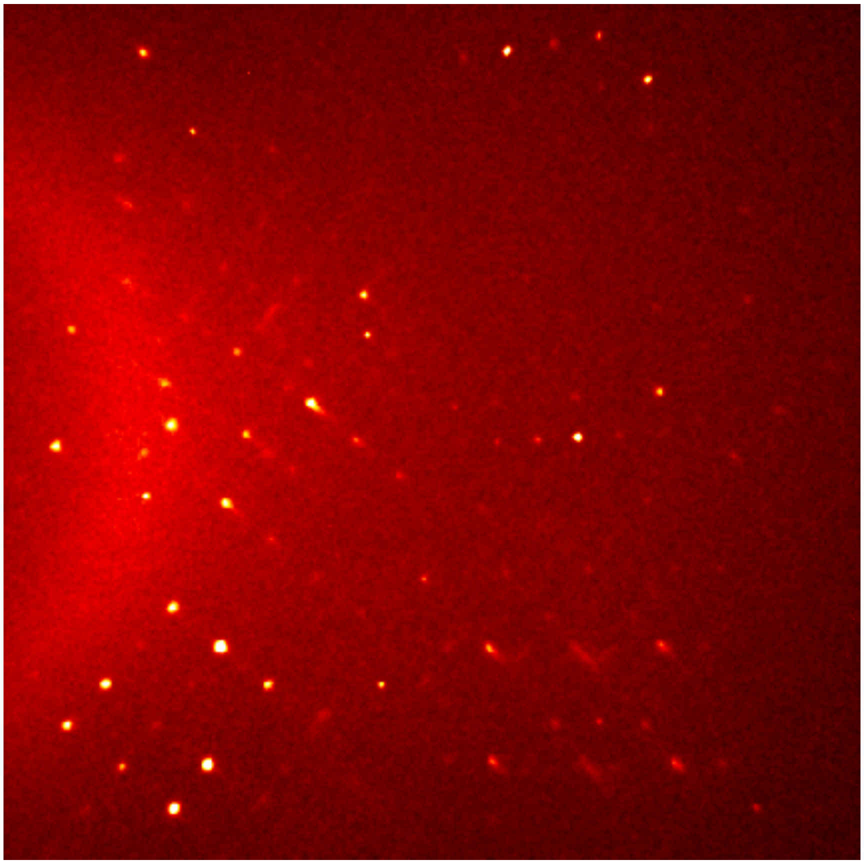

Figure S9 | A characteristic image of the X-ray diffraction data for **1**, illustrating the presence of diffuse scattering along the Bragg peaks due to the inherent disorder of the system. The crystal structures have been modeled to account for this disorder, which gives rise to elongated thermal parameters for some of the atoms.

References

1. D. Feng, W.-C. Chung, Z. Wei, Z. Y. Gu, H.-L. Jiang, Y.-P. Chen, D. J. Darensbourg, H.-C. Zhou, *J. Am. Chem. Soc.*, 2013, **135**, 17105.
2. J. S. Anderson, A. T. Gallagher, J. A. Mason, T. D. Harris, *J. Am. Chem. Soc.*, 2014, **136**, 16489.
3. A. T. Gallagher, M. L. Kelty, J. G. Park, J. S. Anderson, J. A. Mason, J. P. S. Walsh, S. L. Collins, T. D. Harris, *Inorg. Chem. Front.*, 2016, **3**, 536.
4. J. M. Zadrozny, A. T. Gallagher, T. D. Harris, D. E. Freedman, *J. Am. Chem. Soc.*, 2017, **139**, 7089.
5. G. M. Sheldrick, SADABS, version 2.03, Bruker Analytical X-Ray Systems, Madison, WI, 2000.
6. G. M. Sheldrick, SHELXTL, Version 6.12; Bruker Analytical X-ray Systems, Inc.: Madison, WI, 2000.
7. O. V. Dolomanov, L. J. Bourhis, R. J. Gildea, J. A. K. Howard, H. Puschmann, *J. Appl. Crystallogr.*, 2009, **42**, 339.
8. P. Muller, R. Herbst-Irmer, A. Spek, T. Schneider, M. Sawaya, *Crystal Structure Refinement: A Crystallographer's Guide to SHELXL*; OUP Oxford, 2006.
9. W. Morris, B. Volosskiy, S. Demir, F. Gándara, P.L. McGrier, H. Furukawa, D. Cascio, J.F. Stoddart, O.M. Yaghi, *Inorg. Chem.,* 2012, **51**, 6443.
10. C.-M. Lee, C.-H. Chuo, C.-H. Chen, C. C. Hu, M. –H. Chiang, Y.-J. Tseng, C.-H. Hu, G.-H. Lee, *Angew. Chem. Int. Ed.*, 2012, **124**, 5523.
11. (a) J. S. Valentine, *Chem Rev.*, 1973, **73**, 235. (b) L. Vaska, *Acc. Chem. Res.*, 1976, **9**, 175.
12. R. Guilard, M. Fontesse, P. Fournari, *J. Chem. Soc., Chem. Commun.*, 1976, 161.
13. S. Stoll, A. Schweiger, *J. Magn. Reson.*, 2006, **178**, 42
